# Range expansion through fragmented landscapes under a variable climate

**DOI:** 10.1111/ele.12129

**Published:** 2013-05-23

**Authors:** Jonathan Bennie, Jenny A Hodgson, Callum R Lawson, Crispin TR Holloway, David B Roy, Tom Brereton, Chris D Thomas, Robert J Wilson

**Affiliations:** 1 Centre for Ecology and Conservation, University of ExeterCornwall Campus, Penryn, TR10 9EZ, UK; 2 Environment and Sustainability Institute, University of ExeterCornwall Campus, Penryn, TR10 9EZ, UK; 3 Department of Biology, Wentworth Way, University of YorkYork, YO10 5DD, UK; 4 Department of Evolution, Ecology and Behaviour, University of LiverpoolBiosciences Building, Crown Street, Liverpool, L69 7ZB, UK; 5 Department of Animal Ecology, Netherlands Institute of Ecology (NIOO-KNAW)Post Office Box 50, 6700AB, Wageningen, The Netherlands; 6 Sussex Wildlife Trust, Woods MillWest Sussex, Henfield, BN5 9SD, UK; 7 NERC Centre for Ecology & HydrologyMaclean Building, Benson Lane, Crowmarsh Gifford, Wallingford, OX10 8BB, UK; 8 Butterfly ConservationManor Yard, East Lulworth, Wareham, BH20 5QP, UK

**Keywords:** Climate variability, colonisation, environmental threshold model, extinction, habitat networks, mechanistic model, metapopulation dynamics, microrefugia, species distribution

## Abstract

Ecological responses to climate change may depend on complex patterns of variability in weather and local microclimate that overlay global increases in mean temperature. Here, we show that high-resolution temporal and spatial variability in temperature drives the dynamics of range expansion for an exemplar species, the butterfly *Hesperia comma*. Using fine-resolution (5 m) models of vegetation surface microclimate, we estimate the thermal suitability of 906 habitat patches at the species' range margin for 27 years. Population and metapopulation models that incorporate this dynamic microclimate surface improve predictions of observed annual changes to population density and patch occupancy dynamics during the species' range expansion from 1982 to 2009. Our findings reveal how fine-scale, short-term environmental variability drives rates and patterns of range expansion through spatially localised, intermittent episodes of expansion and contraction. Incorporating dynamic microclimates can thus improve models of species range shifts at spatial and temporal scales relevant to conservation interventions.

## Introduction

Climate change is causing shifts in species distributions (Parmesan & Yohe 2003; Chen *et al*. 2011), but limitations remain in our understanding of how climate-driven changes in demographic rates translate into range shifts under realistic conditions of limited dispersal, landscape heterogeneity and environmental stochasticity. Models to predict climate-induced range shifts typically represent spatial climatic gradients as smooth surfaces, and temporal changes either as smooth trends, stepped changes or abrupt shifts from one climate state to another (Guisan & Thuiller 2005; Early & Sax 2011). In contrast, the environmental conditions that affect the survival and fecundity of individuals vary greatly over fine spatial scales, because of variation in topography and habitat structure (Ashcroft *et al*. 2009; Daly *et al*. 2010; Sears *et al*. 2011), and considerable interannual and decadal fluctuations are superimposed upon long-term natural and anthropogenic climate trends (Karl *et al*. 1995; Canning-Clode *et al*. 2011). The dynamics of range shifts are further structured by the spatial distribution of suitable habitat, which is often patchy, and fragmented either naturally or because of anthropogenic processes (Opdam & Wascher 2004). New approaches are needed to understand how habitat fragmentation and spatiotemporal variability in climate combine to determine rates and patterns of climate-driven range shifts (Jackson *et al*. 2009; Bateman *et al*. 2012).

Several approaches have begun to address the effects of fine-scale environmental variability in space and time on species range shifts. Mechanistic models integrate the effects of climate on the ecophysiological processes determining the survival of individuals at their range margins. Such models can incorporate both biophysical models to down-scale regional climate to the operational temperature of organisms, and/or demographic models to up-scale physiological effects to the viability of populations at range margins (Morin *et al*. 2007; Kearney *et al*. 2009; Buckley *et al*. 2010, 2011). At fine spatial resolutions, topography is known to modify temperatures, leading to variability in survivorship (Weiss *et al*. 1988) and local extinction risk (McLaughlin *et al*. 2002). Fine-scale topographic microclimates could thus help species to persist within regions which would otherwise be climatically unsuitable (Ashcroft *et al*. 2009; Daly *et al*. 2010; Sears *et al*. 2011). Temporal variability in climate and extreme weather events have also been statistically linked to historical rates and patterns of range expansion (Gray *et al*. 2006; Walther 2009), and niche models applied to future scenarios of climate change suggest that temporal variability in the climate could determine realised species distributions (Early & Sax 2011). However, the combined effects of short-term, fine-scale variation in climate on population survival and colonisation in fragmented landscapes remain to be incorporated in realistic, empirically tested models of species' range shifts.

Here, we test the hypothesis that fine-scale variation in thermal habitat quality determines the rate and pattern of species range expansion through a fragmented habitat network covering hundreds of kilometres. We develop and test models for the effects of spatial and temporal variation in microclimate on both population and metapopulation dynamics in an exemplar system, the butterfly *H. comma* at its cool range margin in Britain. In common with many other ectotherms (Courtney & Duggan 1983; Kingsolver 1983; Chamaillé-Jammes *et al*. 2006), individuals of this species show thermal constraints on activity and fecundity (Davies *et al*. 2006). We use an empirically derived thermal activity threshold to model the availability, quality and connectivity of suitable habitat patches for the species over 27 years, by applying a high-resolution, physically based model to calculate hourly near-ground temperatures in chalk grassland (Bennie *et al*. 2008). We then investigate the effects of this dynamic thermal environment on range expansion, using two analyses. First, we model annual population size fluctuations as a function of thermal habitat quality in continuously monitored local populations. Second, we let thermal habitat quality determine colonisation potential and extinction risk in a metapopulation model. We calibrate the metapopulation model using patch occupancy observations from 1982 to 1991, and empirically test the model using independent data for the subsequent 18 years (1991–2009). We show that both population and metapopulation models including dynamic microclimate variation: (1) quantitatively out-perform models in which spatial and temporal variability in temperature are not included; and (2) capture important features of the episodic and spatially localised patterns of range expansion, that are important for understanding, predicting and managing the responses of species distributions to climate change.

## Material and Methods

We use three modelling approaches, described in the sections below. First, a topographic microclimate model developed for chalk grassland (Bennie *et al*. 2008) was used to predict thermal habitat quality within all potential habitat patches (see ‘Microclimate model’), providing an input for the other two analyses. Second, we tested the hypothesis that thermal habitat quality determines annual population growth rates and carrying capacity, using annual time series from ten monitoring transects (‘Long-term monitoring transects’; ‘Microclimate and population dynamics’). Third, the influence of microclimate on range expansion was tested by running metapopulation simulations in which patch-level extinction risk and colonisation rates depend on thermal habitat quality (‘Microclimate and metapopulation dynamics’). The metapopulation models were parameterised and independently tested using distribution data from comprehensive field surveys of patch occupancy undertaken at nine-year intervals (‘Distribution survey’).

### Study system

**Figure 1:**
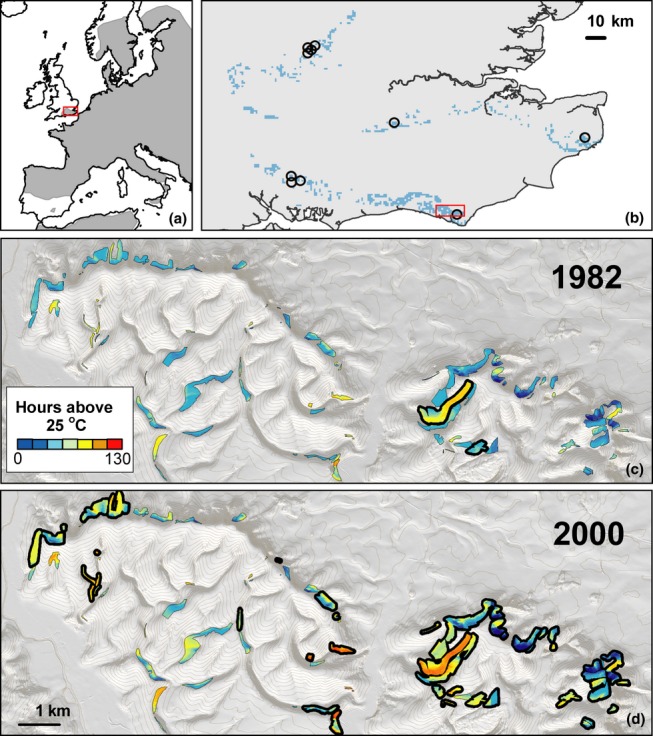
Availability of suitable *Hesperia comma* habitat in SE England: (a) Approximate European distribution of *H. comma* showing the location of the study area; (b) 1 km squares containing suitable habitat (blue), black circles indicate locations of long-term monitoring transects, with red box showing location of 16 × 6 km area enlarged in c and d. (c, d) Example of modelled thermal quality of habitat patches in contrasting years. Colour shading shows the total number of hours in which near-ground temperatures exceeded 25 °C, the critical threshold for activity, during the August flight period in 1982 (c) and 2000 (d), modelled at 5 m resolution. Patches with bold outlines indicate observed presence of *H. comma* in the respective year, showing expansion from a refuge population on a large warm south-facing slope in 1982 to a wider range of patches in 2000 as the number of thermally suitable patches increased.

The silver-spotted skipper *H. comma* (L; Lepidoptera; Hesperiidae) has a holarctic distribution, reaching the north-western limit of its European range in south-east England (Fig. 1a). Here, the species is restricted to grazed calcareous grasslands where its larval food plant, sheep's fescue grass *Festuca ovina* (L.), grows in hot microclimates among short, broken turf (Thomas *et al*. 1986). During the 20th Century, *H. comma* suffered a drastic decline in Britain, attributed to habitat loss caused by agricultural intensification, and changes in chalk grassland habitat due to reduced grazing by European rabbit (*Oryctolagus cuniculus*, L.) following outbreaks of myxomatosis, which led to taller vegetation and a shortage of locations with host plants growing in suitable microclimates. By 1982, fewer than 70 *H. comma* populations survived in Britain, all associated with warm microclimatic conditions on grazed south-facing slopes (Thomas *et al*. 1986). The decline of the species has been followed by a localised re-expansion since 1982 (Thomas & Jones 1993; Davies *et al*. 2005; Lawson *et al*. 2012), associated with the recovery of rabbit populations, widespread adoption of grazing as a conservation management tool, and a series of exceptionally warm summers compared with 20th Century norms (UK Met Office 2011a).

*Hesperia comma* is univoltine, and adult butterflies fly during late summer (August). Adults become fully active (Appendix S1), and egg-laying rates increase rapidly, when temperatures measured close to the ground exceed approximately 25 °C (Davies *et al*. 2006). Recent work has shown that (1) the species occurs in higher densities on south and west facing slopes where this temperature threshold is most frequently exceeded during the flight season; and (2) the risk of local population extinction at this upper-latitude range margin is significantly higher on cooler, shaded north-facing slopes (Lawson *et al*. 2012).

### Distribution survey

Comprehensive field surveys to record the distributions of *H. comma* and its habitat were conducted at nine-year intervals from 1982 to 2009. Suitable habitat patches included any agriculturally unimproved chalk grassland containing more than 5% cover of the larval food plant *F. ovina*. The surveys covered five main habitat networks, each of which supported refuge populations in 1982 (Fig. 1). One network, the Chiltern Hills in the north–west of the region, was not surveyed in 1991. In surveys prior to 2009, all habitat patches identified within 20 km of existing records of *H. comma* were surveyed for presence of adult butterflies and/or eggs (Thomas *et al*. 1986; Thomas & Jones 1993; Davies *et al*. 2005). In 2009, to ensure long-distance dispersal events were not missed and to provide full coverage of the available habitat network, all chalk grassland patches within 30 km of an existing distribution record of the species (including records held by the national UK butterfly recording scheme) were visited, and mapped using handheld GPS (Lawson *et al*. 2012). The 906 habitat patches that were recorded, ranging in size from 39 m^2^ to 71 ha (mean 3.61 ha), form the habitat network for the microclimate and metapopulation models.

### Long-term monitoring transects

Population dynamic data were analysed for *H. comma* populations that are regularly surveyed by the United Kingdom Butterfly Monitoring Scheme (UKBMS). In this scheme, transect counts are carried out weekly at fixed locations, provided that weather conditions meet specified criteria (Pollard & Yates 1993). We limited our analysis to the 10 UKBMS transects having positive counts of *H. comma* for at least ten consecutive years between 1982 and 2009 (circles in Fig. 1b; details in Table S1). These transects include three north-facing, six south- or south-west- facing, and one flat habitat patch, and are located across all five main habitat networks. While UKBMS transects often pass through several habitat types, only data from the transect section(s) covering suitable habitat for *H. comma*, as defined above, were used in this study. The sampling units, whether full UKBMS transects within chalk grassland, or sections of longer transects through mixed habitat, are henceforth referred to as ‘transects’. An annual index of abundance for each transect was calculated by the addition of weekly counts, accounting for missing values (Moss & Pollard 1993), and this index was divided by the length of the transect to yield a relative density estimate *D* for each year. The number of consecutive years with data varied between transects, but the ten transects selected gave a sample size of 191 estimates of interannual changes in population density (Table S1).

### Microclimate model

We used a spatially explicit model of topographic microclimate which was developed and empirically tested in typical *H. comma* chalk grassland habitat (see Bennie *et al*. 2008 for details), to estimate interannual variation in the thermal niche of *H. comma* at 5 m resolution within each habitat patch. This microclimate model estimates hourly air temperature at 10 cm height in short turf, allowing for changes in solar altitude and azimuth throughout the day, given slope, aspect, topographic shading, hourly radiation balance, ambient air temperature and wind speed. Near-surface air temperatures within the grassland sward were modelled, rather than the operative temperature of the butterfly itself, for comparison with existing data from field observations on activity (Appendix S1; Davies *et al*. 2006). We modelled temperature during the flight season, rather than at other stages of the species' annual life-cycle, because preliminary analysis suggested strong thermal constraints on population growth during this period, with limited effects of climatic variables at other times of year (Appendix S1).

Regional meteorological data were used to provide hourly estimates of ambient meteorological conditions for input to the microclimate model. A time series of hourly air temperature, wind speed and sunshine hours from 1982 to 2009, representative of weather patterns in south-east England, was obtained from Heathrow Airport (UK Met Office 2011b), located between the five habitat networks (51°28′ N 0°27′ W). To allow for regional gradients in ambient climate between Heathrow and the habitat patches, the hourly time series was adjusted for consistency with a 5 km resolution gridded observation data set (Perry & Hollis 2005). This data set included daily maximum and minimum temperature values, and monthly mean wind speed and monthly mean sunshine duration. For each 5 km grid cell, we linearly re-scaled Heathrow's hourly temperature for each day so that daily maximum and minimum temperatures matched the gridded data. We also re-scaled hourly wind speed and sunshine hours so that monthly average values matched the gridded monthly averages, constraining sunshine duration in each hourly timestep to be ≤ 1 h.

The microclimate model was run using topography derived from a 5 m resolution digital terrain model (Intermap Technologies 2007). The radiation balance for each cell was calculated by adjusting clear-sky direct and diffuse radiation for sunshine hours, slope, aspect and topographic shading (Bennie *et al*. 2008, 2010). Local wind speed was adjusted for topographic shelter using a shelter index derived from the tangent of the angle to the horizon in the direction of the wind (Ryan 1977). The microclimate model incorporates the main factors determining daytime near-surface temperatures in summer in this system. Other significant topographic effects on temperature, such as temperature inversions, were not modelled, as their influence is limited to night-time and winter periods when long-wave radiation dominates the energy balance (Bennie *et al*. 2010).

The modelled hourly microclimate temperature at 5-m resolution was converted into an annual estimate of thermal habitat quality for *H. comma* in each of the 906 surveyed habitat patches. Thermal habitat quality was obtained as the sum of the mean number of hours that each 5-m cell in the habitat patch exceeded a temperature threshold of 25 °C, during August of each year. This threshold represents the low-temperature threshold for activity and egg-laying in this species (Appendix S1; Davies *et al*. 2006).

### Microclimate and population dynamics

To test the influence of annual climatic and microclimatic variability on population growth rates measured in the field, we modelled annual density on each of the 10 long-term monitoring transects (Table S1). Population time series were fitted with discrete-time logistic models of population growth, Δ*D* = *rD* (1−*D*/*K* ) in which both the intrinsic rate of population growth *r* and carrying capacity *K* vary as linear functions of temperature during the flight season. Models were fitted to observed values of Δ*D* using the lmer function in the lme4 package of R version 2.13.0 (R Development Core Team 2011), and model performance was assessed using the Akaike's Information Criterion (AIC). Transforming the above equation, the fitted model form was


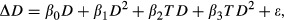


where *T* is one of several measures of temperature (see below) relevant to the transect, *ε* is the error term, and *β*_0_, *β*_1_, *β*_2_ and *β*_3_ are fitted parameters – *β*_0_ and *β*_2_ represent the slope and intercept of the linear dependency of *r* on *T*, and *β*_1_ and *β*_3_ represent the slope and intercept of the linear dependency of *−r/K* on *T*.

To test the usefulness of microclimate for modelling population dynamics, values of *T* were randomised between all transects and years, models were fitted for observed population dynamics, and fit values (*R*^2^) were calculated. This process was repeated 10 000 times, and the *R*^2^ for observed *T* was compared with the distribution to obtain a *P*-value.

To test whether a population density model incorporating modelled patch microclimate, rather than regional climate, better described the relationship between temperature and population dynamics, we tested fit to observed changes in population density of the following models, each representing different hypotheses about how temperature influences intrinsic growth rates and carrying capacity. The model forms were as follows: (i) *r* and *K* are equal across all transects, and for all years, representing no climatic or transect-specific influence on population dynamics (‘Single fitted *r* and *K*’); (ii) *r* and *K* vary between transects (as a random factor) with no relationship with temperature, representing transect-specific population dynamics with no influence of climate (‘Fitted *r* and *K* varying between transects’); (iii) *r* and *K* vary linearly with the August mean daily maximum temperature from gridded climate data at 5 km resolution (Perry & Hollis 2005), with no effect of transect (‘Regional climate’); (iv) *r* and *K* vary with regional (5 km) temperature as above, with transect included as a random factor determining the intercept, allowing *r* and *K* to vary according to transect-level differences (‘Regional climate, intercepts varying between transects’); (v) *r* and *K* vary with regional temperature as above, with transect included as a random factor determining both the intercept and slope of this relationship (‘Regional climate, slopes and intercepts varying between transects’); (vi) *r* and *K* were fitted across all ten transects using modelled thermal habitat quality for each year, such that *r* and *K* were determined by interactions between annual temperature, sunshine, wind speed and topography (‘Microclimate’); (vii) a model with the same structure as model (vi), using thermal habitat quality from the previous year.

### Microclimate and metapopulation dynamics

To predict regional range expansion, we modelled *H. comma* metapopulation dynamics using the incidence function model (IFM; Hanski 1994). In the IFM, the probability of colonisation in a given timestep is *C*_*i*_ = *S*_*i*_^2^/(*y* + *S*_*i*_^2^) and the probability of extinction is *E*_*i*_ = (1−*C*_*i*_)*μA*_*i*_^−x^, where *S*_*i*_ denotes the connectivity of patch *i*. Connectivity is given by *S*_i_ = sum_*j*_[(*d*_*ij*_ + 0.05)^−*α*^*A*_*j*_^b^], and *A*_*i*_ is a proxy for the population carrying capacity of patch *i*, usually taken as the patch area, influencing both local extinction risk and the capacity to generate colonists (Moilanen & Hanski 1998). In this model, *d*_*ij*_ is the centre-to-centre distance between each pair of patches *i* and *j*. The parameters (*α, y, μ, x,* and *b*) are used to relate the size and configuration of patches and populations to dispersal (*b*, *α*), colonisation probability (*y*), and extinction risk (*μ, x*). We used a power law dispersal kernel (*α*) because of its better fit than negative exponential kernels to mark-recapture data for *H. comma* (Hill *et al*. 1996).

For our comparison of models excluding or including microclimate, *A*_*i*_ was either the polygon area of patch *i* (‘habitat area model’), or the patch area weighted by the modelled thermal habitat quality (number of hours each August exceeding the thermal threshold), scaled such that the mean value of thermal habitat quality across patches between 1982 and 1991 equals 1 (‘microclimate model’).

We used nonlinear optimisation in the R package bblme (Bolker & R Development Core Team 2010) to fit the five IFM parameters (*α, y, μ, x* and *b*) using survey data from the 686 patches for which occupancy data (population presence or absence) were available in 1982 and 1991, and maximising the likelihood of the 1991 occupancy given the 1982 occupancy. Confidence intervals on parameter estimates were wide, so for simulations we sampled parameter sets in proportion to their likelihood, within the joint 95% confidence interval. The parameter estimates and confidence intervals were very similar whether *A*_*i*_ was given as polygon area or thermal habitat quality adjusted area, but nevertheless when simulating we used the parameters that had been fitted using the appropriate measure of *A*_*i*_. We ran 500 iterations of each fitted IFM for all 906 habitat patches recorded in 2009, either starting from the 1982 observed distribution, and evaluating fit to the observed 2000 distribution (model run 1), or starting from the 2000 observed distribution and evaluating fit to the observed 2009 distribution (model run 2).

In our empirical tests of metapopulation simulations, the likelihood of each presence or absence observation in 2000 or 2009 was calculated as the proportion of model runs in which that observation was reproduced in the simulation. Overall, AIC was calculated using these likelihood values, either treating every patch as an independent observation, or grouping patches within 5 km squares, and evaluating the joint likelihood of every patch in a square being predicted correctly by the simulation. The latter is a conservative method to account for spatial autocorrelation of patch occupancy. The importance of microclimate in determining the spatial pattern of range expansion was tested by comparing AIC values between simulations using the microclimate model *versus* the habitat area models.

## Results

### Microclimate and population dynamics

In the ‘microclimate’ model (vi) where transect- and year-specific microclimate determined local population growth rates, all fitted parameters were significantly different from zero at *P* < 0.001 (*β*_0_ = 1.2 × 10^−6^, *β*_1_ = 1.8 × 10^−6^, *β*_2_ = 5.2 × 10^−7^, *β*_3_ = 8.7 × 10^−5^). The *R*^2^ value of 0.15 was significantly greater than expected (randomisation test, *P* = 0.02). The microclimate model (model vi) had more empirical support than any of the alternative models for observed population dynamics at the ten continuously monitored transects (Table 1). The next best performing models incorporated the effects of regional climate only (model iii), or regional climate interacting with random transect-specific effects (model v), reinforcing the importance of interactions between interannual climatic variability and local habitat characteristics (including topography) in driving population dynamics in this system. Model v was able to fit observed data reasonably well (*R*^2^ = 0.314), but had a high number of parameters due to the inclusion of transect as a random effect.

**Table 1:** Models for the population dynamics of *Hesperia comma* on regularly monitored transects between 1982 and 2009. Models represent change in density *D* between years and take the form Δ*D* = *rD*(1−*D/K* ), where *r* is the intrinsic rate of population growth and *K* is carrying capacity. *N* = 191 time step per transect observations; see Table S1 for transect details, and methods text for model descriptions

Model	Parameters	*R*^2^	AIC	ΔAIC
vi) Microclimate	4	0.150	−652.0	0
iii) Regional climate	4	0.093	−639.8	12.2
v) Regional climate, slopes and intercepts vary between transects	40	0.314	−638.2	13.8
i) Single fitted *r* and *K* for all transects	2	0.085	−638.1	13.9
vii) Microclimate for previous year	4	0.067	−633.5	18.5
ii) Fitted *r* and *K* varying between transects	20	0.134	−612.1	39.9
iv) Regional climate, intercepts vary between transects	22	0.124	−610.0	42.0

### Microclimate and metapopulation dynamics

**Figure 2:**
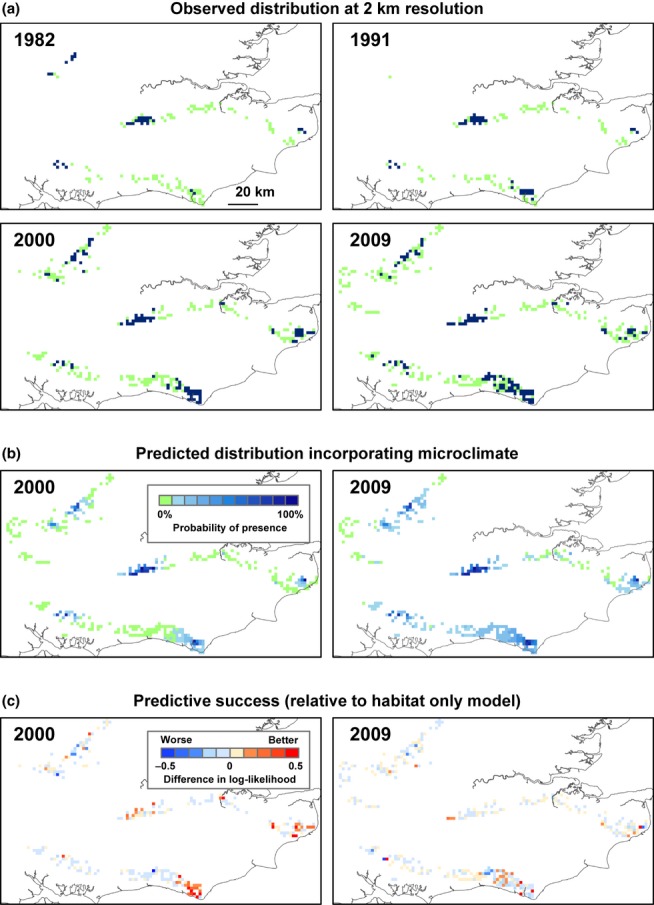
Observed and predicted range expansion of *Hesperia comma* from 1982 to 2009. The observed distribution maps (a) show 2 km grid cells containing surveyed habitat occupied by *H. comma* (blue) or vacant (green). Only comprehensively surveyed cells are coloured in each survey date. Predicted distribution maps (b) show the modelled patch network aggregated to 2 km resolution, in each case shaded to represent the proportion of 500 model runs in which each grid square was occupied in the microclimate model. Predictive success (c) is shown relative to the habitat area model, showing the difference in log-likelihood achieved by incorporating microclimate.

**Figure 3:**
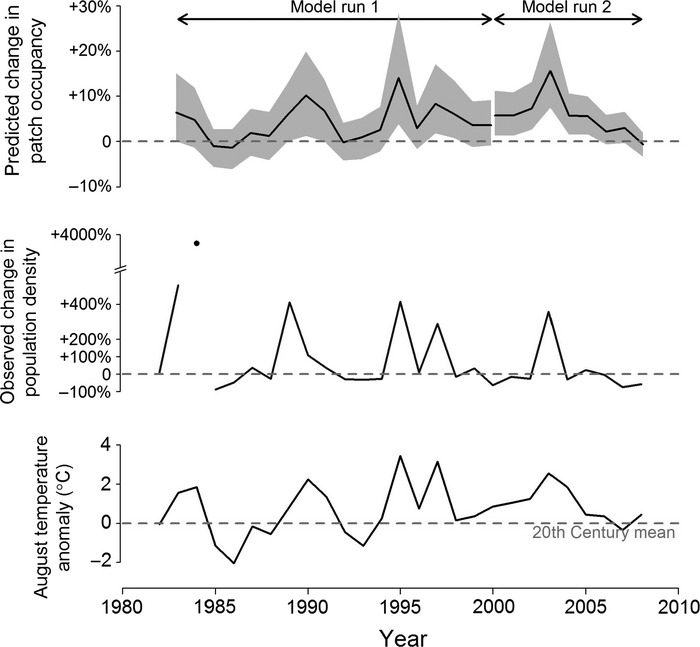
Changes in patch occupancy at the range margin of *Hesperia comma* predicted by the microclimate model. Top panel shows median value of 500 simulations, shaded area shows 95 percentile limits. Colonisations greatly exceed extinctions in warm years. Since the mid-1980s, this pattern closely matches the trend shown by changes in population density based on estimates from transect surveys (mid panel; plot shows annual rate of change) and the August temperature record for south-east England (lower panel).

The ‘microclimate’ model captures the observed rate and pattern of range expansion by the species from 1982 to 2000, and from 2000 to 2009 (Fig. 2), and has consistently more support than the ‘habitat area’ model in predicting the occupancy state (presence vs. absence) of individual habitat patches in the test years, for both model runs (overall ΔAIC *c*. 40 for 2000 and 2009: Table 2, Fig. 2). In terms of the temporal pattern of range expansion, while the habitat area model predicted a steady annual increase in patch occupancy by around 3% each year, the ‘microclimate’ model predicted sharp peaks in colonisation during warm summers, counteracted by no net colonisation or even an excess of extinction over colonisation in cold summers (Fig. 3). The pattern of peaks and troughs in relative numbers of colonisations and extinctions correlates well with the observed population dynamics of *H. comma* in the independent data set of populations that have been continuously monitored by the UK Butterfly Monitoring Scheme since 1985 (Fig. 3; Pearson's *R*^2^ = 0.52, *P* < 0.001).

**Table 2:** Performance of microclimate- vs. habitat area-based metapopulation models in predicting observed occupancy of habitat patches by *Hesperia comma* in two time periods. 500 simulations were run for the periods 1982–2000 and 2000–2009, starting with observed occupancy. Table shows the Akaike Information Criterion (AIC) based on the log-likelihood that observed occupancy was correctly predicted a) for individual habitat patches (612 in 2000; 906 in 2009), or b) for all habitat patches grouped per 5 km square in which they occur (*n* = 115 × 5 km squares in 2000, 149 in 2009). b) represents a more conservative measure which takes account of likely spatial autocorrelation of nearby patches. ΔAIC is positive where the microclimate model out-performed the area model

Test data	Start year	1982	2000
	End year	2000	2009
a) Independent patches	AIC Habitat area model	728.2	841.4
	AIC Microclimate model	688.6	799.1
	ΔAIC	39.5	42.3
b) All patches per 5 km square	AIC Habitat area model	440.8	550.9
	AIC Microclimate model	434.9	535.5
	ΔAIC	5.8	15.4

The 9-year test periods following the model calibration period, from 1991 to 2000 and 2000 to 2009, presented contrasting thermal conditions, and different rates and patterns of range expansion. During the warmer 9-year period simulated in model run 1 (1991–2000), *H. comma* rapidly expanded its distribution in Britain: 156 unoccupied habitat patches were colonised, but only 4 of 78 local populations recorded in 1991 were extinct in 2000. The microclimate model was more successful than the habitat area model in predicting long-distance colonisations of relatively high quality patches, with improved fit for large, relatively warm patches which were vacant and isolated from occupied patches in 1982 but occupied in 2000 (Fig. S1).

Conditions were on average slightly cooler between 2000 and 2009 (model run 2), albeit warmer than the long-term average (Fig. 3). In this second test period, 67 new populations were recorded, and the range of the species locally expanded up to 18 km (Fig. 2), but the colonisations were set against 48 local extinctions. The greater support for the microclimate vs. habitat area model during the second period (Table 2) appears mainly due to more accurate prediction of absence of the species caused by local extinctions from micro-climatically cool habitat patches (Fig. S1). In contrast, the habitat area model was somewhat more successful than the microclimate model in identifying relatively isolated habitat patches that were occupied by the species in 2009 (Fig. S1).

## Discussion

We integrate fine-scale spatial and temporal variability in weather and microclimate with the realistic representation of a fragmented habitat network, to simulate the range expansion of a thermally limited butterfly species. Our empirical tests of this model, using detailed observations of population dynamics and patch occupancy over 27 years, support the hypothesis that fine-scale variation in thermal habitat quality determines the rate and extent of range expansion at the species' cool margin during climate warming.

### Climate variability and population dynamics at range margins

At species' range margins, demographic variability is often linked to fine-scale spatial and temporal variation in climatic factors (Kingsolver 1983; Roy & Thomas 2003). As a result, the geographical range margins of species may be characterised by high demographic variability (Thomas *et al*. 1994; Curnutt *et al*. 1996), and climate warming has led to a dampening over time in the population variability of species near their poleward range margins (Oliver *et al*. 2012). Our findings confirm for the butterfly *H. comma* that population variability can be linked explicitly to spatial and temporal variation in thermal habitat quality, which is a function of both weather conditions and the topographic characteristics of different habitat patches, particularly aspect (Table 1).

Local demographic variability in response to environmental stochasticity has important implications for the rate and extent of range expansion for species in which a discontinuous distribution of habitat creates a metapopulation structure (Opdam & Wascher 2004). By including the effects of temporal variation in microclimate, our metapopulation model captures the episodic nature of range expansion by *H. comma* (Fig. 3), predicting enhanced rates of colonisation during hotter years that favour population growth, and local extinctions during cool periods. During hot years, the microclimate-driven metapopulation model predicts net peaks of colonisation over extinction. The timing of these peaks coincides with the recorded population dynamics of the species from independently monitored populations in Britain (Fig. 3), demonstrating how the combined microclimate and metapopulation model is able to translate local demographic effects of climate variability to the temporal pattern of range expansion by the species.

### The spatial and temporal resolution of range shifts

Evidence from palaeoecology suggests that the existence of microrefugia may be necessary to explain the responses of species' geographical ranges to climate change (Sublette Mosblech *et al*. 2011). In addition, temporal variability in climate can influence the location of species range margins (Early & Sax 2011; Bateman *et al*. 2012) by driving episodes of colonisation, population growth and local extinction (Jackson *et al*. 2009; Walther 2009). Here, we confirm empirically that topographic variation at fine scales plays an important role in determining rates and patterns of range expansion under contemporary climate change and variability. As a species shifts its range, topographic variation may provide climatically favourable microrefugia beyond the more continuous geographical range limits. For *H. comma*, warm, south-facing slopes act as nuclei for range expansion by increasing the chances of colonisation and population growth in favourable (hot) years at the cool range margin; but importantly, these warm environments may also enable the species to retain footholds at the margins of the distribution during less favourable (cool) years.

Our results show that including the effects of temporal and spatial variability in climate can improve the ability of a metapopulation model to capture the observed spatial dynamics of range expansion. Our empirical data set encompasses a period of range expansion containing the three warmest Augusts recorded in south-east England (1995, 1997 and 2003) since at least 1910 (UK Met Office 2011b). Incorporating microclimate into the metapopulation model helped to identify the high thermal-quality but geographically isolated habitat patches that were colonised by *H. comma* during a run of hot years between 1991 and 2000. The metapopulation model incorporating spatial and temporal variation in climate also identified more accurately the microclimatically marginal habitat patches (e.g. on north-facing slopes; Lawson *et al*. 2012) from which the species suffered local extinction during the cooler, second period of metapopulation simulation (2000–2009). These empirically tested simulations confirm how advances made by a thermophilic species in unusually warm seasons can be offset by limited expansion or retraction to locations with hot microclimates during cooler periods, from which they may expand again during subsequent warmer seasons. In the case of *H. comma*, an increased frequency of hot summers during the predicted continued warming trend (Solomon *et al*. 2007) is likely to cause future pulses of population growth, which will translate into intermittent waves of colonisation of habitats beyond the species' current range limits. As such, future rates of range expansion are likely to depend on the frequency and magnitude of temporal fluctuations in climate, in addition to changes in mean climate.

### Mechanistic models of range shifts under climate variability

Species distribution modelling has moved in recent years away from purely correlative models towards more mechanistic approaches (Morin *et al*. 2007; Buckley *et al*. 2010, 2011). Such mechanistic approaches typically involve integrating models and data across drastically different scales. Available climate data must be down-scaled to biologically relevant spatial and temporal scales, and then biological responses at the level of the organism or population must be up-scaled to predictions of distribution at a regional or even global scale. Incorporating fine-scale effects of climate may require a flexible approach for different species and landscapes, which may be deduced from direct activity and physiological measurements (as here) or from statistical relationships between species and environment (Sears *et al*. 2011). Our results demonstrate how incorporating both climatic variability and realistic representations of landscape structure, including topography, into down-scaled climate, can capture potential effects of microclimate in driving population-level responses. Furthermore, we suggest that representation of both population- and metapopulation-level processes may need to be incorporated into methods to translate the effects of climate on individuals to species range dynamics, including both extinction and colonisation at range edges.

### Implications for conservation

For species experiencing climate variability around the warming trend, range expansion models that incorporate dynamic microclimates and realistic conditions of habitat fragmentation have the potential to reveal the dynamics of colonisation and extinction at spatial and temporal scales relevant to conservation interventions. It may already be feasible for some species of conservation concern to predict the success of assisted colonisation schemes given different initial introduction sites, or the ability of fragmented habitat networks to support range shifts; however, given that different species and their habitats will respond in different ways and on different timescales to climate change, it will remain a challenge to give specific and dependable conservation advice for multiple species. Nevertheless, there is a need to develop principles of action, and test their effectiveness through realistic models and long-term observations where available. One strategy for conservation of biodiversity in the face of uncertain species responses to climate is to focus conservation efforts on retaining landscape structures (including geological and topographic units) that will promote diversity and facilitate range shifts (Beier & Brost 2009; Anderson & Ferree 2010). Hence, when planning regional conservation for a suite of species, it may be important to protect and manage suitable habitats that include a range of microclimates. The warmest habitats represent potential footholds for species expanding their distributions polewards, whereas the coolest locations may act as microrefugia for species at ‘warm’, lower latitude range margins. More generally, topographic and microclimatic variability could enhance metapopulation persistence by increasing asynchrony in local population dynamics. Although the broader geographical direction of range shifts may be deduced from species-environment relationships and bioclimate modelling (e.g. Early & Sax 2011), our results show that conservation planning in specific regions or landscapes may benefit from an understanding of the effects of local variation in microclimate on the likelihood of metapopulation persistence or expansion.
