# Supplementary material for: Range expansion through fragmented landscapes under a variable climate
Source: Ecol Lett. 2013 May 23;16(7):921–9. doi: 10.1111/ele.12129 (PMC3738923; doi:10.1111/ele.12129)
Supplement: Supplementary file 1 [file ele0016-0921-SD1.docx]

**Supporting Information**

Appendix S1. Estimation of thermal habitat quality

Climate and weather events may affect demographic rates in insects at different stages of their life-cycle, and hence identifying the key life stages that constrain population growth rates of thermally constrained species at a particular range margin is critical (Radchuk *et al.* 2013). We directly tested for population-level effects of temperature and precipitation on the log-transformed national-level population index of the species using a first-order autoregression following Roy *et al.* (2001), using monthly mean temperature and total precipitation data for each month of the 24 months prior to, and including, August of the survey year using regional climate data (UK Met Office 2011).

We modelled the logged national population index as a first-order autoregressive model with weather variables as covariates. Models were fitted to observed values of Δ*D* using the glm function in the lme4 package of R version 2.13.0 (R Development Core Team 2011), and model performance was assessed using the Akaike’s Information Criterion (AIC; Burnham & Anderson 2002). August temperature far outperformed any other monthly climatic variable, giving the lowest AIC value, and a difference in (∆AIC) of 8.3 compared with the next best model. This is consistent with our observations that observed butterfly numbers (and subsequent egg counts) are closely linked to the weather during the flight season. South-east England has a relatively mild winter climate compared to more continental areas of *H. comma*’s distribution, making it unlikely that low temperature effects on winter survival rates constrain population growth in the region. Hence, we confined our modelling of microclimate effects to August temperature, assuming that climatic variation at other stages of the butterfly’s life cycle was of secondary importance in driving population dynamics in this region during the period of interest. We note that in other regions, and potentially under future climate conditions, life-stages other than the adult stage may constrain populations of this species.

Previous work suggests that activity and egg-laying by adult female *H. comma* in Britain shows a marked increase at near-ground temperatures exceeding 25°C (Davies *et al.* 2006). To confirm the influence of environmental temperature on activity, we conducted 100 m long transects to record the proportion of individuals that were active, at Malling Down, Sussex, UK (50° 52' N 0° 01' E) each day from 8-12 August 2010, between 1000 and 1700 h British Summer Time. Activity was recorded on 110 transect sections in total. Butterflies flying or engaged in courtship were classified as active; butterflies resting or basking were classified as inactive. Temperatures were measured from miniature dataloggers with external probes mounted at 10 cm height (Tinytalk Tag 2 TK-4023, Gemini Dataloggers, Chichester, UK) on the same hillside at the time that the transects were walked. We used logistic regression in R (R Development Core Team 2011) to model the proportion of active butterflies as a function of environmental temperature. The proportion of active individuals showed a best-fitting model (n = 110 transects, brackets indicate SE) of logit (P) = -2.61 (±0.53) + 0.111 (±0.020) × T, where P is the proportion of observed individuals that were active, T is the mean temperature during the transect walk (°C at 10 cm height), and logit represents the logistic function. This model has ∆AIC of 33.4 compared to the null model, and gives a threshold for 50% activity at 23.7°C (95% confidence interval 21.2 to 25.3 °C).

Our choice of a single absolute ambient temperature threshold for activity (25 °C) is clearly a simplification of the thermal ecology of the species, as is the assumption of a simple linear relationship between the time available for activity and both within-patch population growth rate and carrying capacity. However, we found that the modelled duration above a range of temperature thresholds between 20 and 30 °C was highly correlated, and no evidence for inhibition of the population response to temperature during the hottest observed summers in the UK, so we infer that our results will be robust to the choice of threshold and response forms.

Table S1. UK Butterfly Monitoring Scheme transects used for population modelling. Transect name, number of years between 1982 and 2009 in which positive counts were recorded in both the current and previous year, and the compass direction of the aspect of the transect are shown.

| **Transect** | **Location** | **Number of years** | **Aspect** |
| --- | --- | --- | --- |
| Aston Rowant N (i) | 51º 40’N 0 º 57’W | 23 | S |
| Aston Rowant N (ii) | 51º 40’N 0 º 57’W | 19 | N |
| Aston Rowant S (i) | 51º 39’N 0 º 57’W | 18 | S |
| Aston Rowant S (ii) | 51º 39’N 0 º 57’W | 21 | N |
| Beacon Hill (i) | 51º 39’N 0 º 57’W | 23 | S |
| Beacon Hill (ii) | 51º 39’N 0 º 57’E | 18 | N |
| Lydden Down | 51º 10’N 1 º 15’E | 11 | SW |
| Old Winchester Hill | 50º 59’N 1 º 05’W | 18 | S |
| Lullington Heath | 50º 47’N 0 º 11’E | 24 | Flat |
| Box Hill Zig Zag | 51º 15’N 0 º 19’W | 16 | S |

**Figure S1.** Differences in fit between the microclimate model and the habitat area model as a function of patch-specific attributes. Difference in fit is measured as the difference in the log likelihood of the observed occupancy of each patch in 2000 (upper panels) or 2009 (lower panels). Positive values of the difference indicate that the microclimate model fit better, and vice versa. Patches are classified by whether or not they were occupied during the test year (end of the simulation: black boxplots for empty, red boxplots for occupied) and by either patch microclimate quality (minimum quality during the simulation period), patch isolation (distance from the nearest occupied patch at the start of the simulation) or patch area. The width of each boxplot is proportional to the square root of the number of observations in that category. Boxes show the quartiles and the whiskers extend to the maximum and minimum.
